# A proteomic map of thromboinflammatory signatures in antiphospholipid syndrome: results from antiphospholipid syndrome alliance for clinical trials and international networking (APS ACTION) registry

**DOI:** 10.3389/fimmu.2025.1676578

**Published:** 2025-10-16

**Authors:** Alexander Pine, Ayesha Butt, Laura Andreoli, Jason S. Knight, Maria Gerosa, Irene Cecchi, D. Ware Branch, Rosario Lopez-Pedrera, H. Michael Belmont, Nina Kello, Michelle Petri, Ricard Cervera, Vittorio Pengo, Pier Luigi Meroni, Hannah Cohen, Rohan Willis, Maria Laura Bertolaccini, George Goshua, Sean Gu, John Hwa, Alfred I. Lee, Doruk Erkan, Anish V. Sharda

**Affiliations:** 1 Section of Hematology, Yale University School of Medicine, New Haven, CT, United States; 2 University of Brescia, Brescia, Italy; 3 University of Michigan, Ann Arbor, MI, United States; 4 University of Milan, Milan, Italy; 5 University of Turin, Turin, Italy; 6 University of Utah and Intermountain Healthcare, Salt Lake, UT, United States; 7 Maimonides Institute of Biomedical Research of Cordoba, Córdoba, Spain; 8 New York University Langone Medical Center, New York, NY, United States; 9 Northwell Health, New Hyde Park, NY, United States; 10 Johns Hopkins University School of Medicine, Baltimore, MD, United States; 11 Department of Autoimmune Diseases, Hospital Clínic, Institut d'Investigacions Biomèdiques August Pi i Sunyer (IDIBAPS), University of Barcelona, Barcelona, Catalonia, Spain; 12 University of Padova, Padova, Italy; 13 IRCCS Istituto Auxologico Italiano, Milan, Italy; 14 University College London, London, United Kingdom; 15 University of Texas Medical, Galveston, TX, United States; 16 St Thomas’ Hospital, London, United Kingdom; 17 Yale Cardiovascular Research Center, Yale University School of Medicine, New Haven, CT, United States; 18 Hospital for Special Surgery and Weill Cornell Medicine, New York, NY, United States

**Keywords:** antiphospholid syndrome, plasma proteomics, thromboinflammation, immune activation, coagulation, complement

## Abstract

**Introduction:**

Antiphospholipid syndrome (APS) is an autoimmune disease with thromboembolic and obstetric morbidity arising via a model of immunothrombosis. Individuals with APS may present with thrombotic (TAPS), obstetric (OAPS), or microvascular (MAPS) disease, while many have circulating antiphospholipid antibodies (aPL) without APS classification (NoAPS). Multiple pathophysiologic mechanisms have been proposed in APS, including activation by aPL of platelets, endothelial and immune cells, as well as complement and coagulation pathways; however, the pathophysiology of APS, particularly transition of clinical APS from aPL remains unclear.

**Methods:**

Seeking to define the inflammatory signature of APS, we carried out an unbiased proteomic screen of persistently aPL-positive patients with different clinical phenotypes from the international APS Alliance for Clinical Trials and International Networking (ACTION) Registry and compared them to 10 healthy controls. 6398 unique proteins were estimated using an DNA aptamer-based assay. Subsequently, we validated our findings in 34 additional patients.

**Results:**

Our data show that the mere presence of aPL confers a distinct thromboinflammatory signature characterized by the activation of coagulation, complement, innate and adaptive immune response pathways shared by all APS subtypes. Pathway enrichment analysis revealed increasing enrichment with rising statistical significance of thrombosis, complement, neutrophil and other innate and adaptive immune activation, as well as extracellular matrix (ECM) organization with increasing clinical severity, suggesting a model of progressive thromboinflammation in evolution of APS from NoAPS to TAPS and MAPS.

**Conclusions:**

Our findings provide novel insights into the pathogenesis of APS and identify potential novel targets for diagnostic and therapeutic intervention in APS across its entire spectrum.

## Introduction

Antiphospholipid syndrome (APS) is an autoimmune disease characterized by the presence of circulating antiphospholipid antibodies (aPL), such as anticardiolipin and anti-β_2_-glycoprotein 1 (anti-β_2_ GP1) antibodies, and associated with thromboembolic disease and obstetrical morbidity (1, 2). A striking feature of APS is the propensity to both arterial and venous thromboembolic disease, as well as thrombosis of the microcirculation (1, 2). The resulting thrombotic complications, particularly strokes, myocardial infarction, venous thromboembolism and microvascular thrombi of the skin, lungs, and kidneys, are associated with increased morbidity and mortality (1, 2). Obstetric morbidity in APS manifests as placental insufficiency, recurrent miscarriages, fetal demise, and preeclampsia/eclampsia (1). APS is sometimes associated with other autoimmune diseases, particularly systemic lupus erythematosus, but can occur as a primary syndrome (1, 2). The clinical spectrum of APS can range from individuals with circulating aPL but no thromboembolic/obstetric disease (NoAPS), to those with moderate-to-large vessel thrombosis (TAPS), pregnancy morbidity (OAPS), and/or microvascular disease (MAPS) (e.g., skin necrosis, aPL-nephropathy or diffuse alveolar hemorrhage) (1, 3). While the clinical manifestations of APS are well-recognized, molecular mechanisms driving antibody-induced thrombosis and obstetric morbidity remain incompletely understood. As a result, other than anticoagulation, therapeutic strategies in APS are currently limited.

Although the presence of aPL are necessary for the development of thrombosis, what precipitates thromboembolic disease remains unknown (2). As such, a ‘two-hit’ concept of APS has been proposed, which hypothesizes that vascular injury or inflammatory stimulus (second hit) triggers thrombosis in a generalized procoagulant state created by aPL (first hit) (4). The pathophysiologic mechanisms distinguishing different APS subtypes also remain unknown. aPL interact with phospholipid binding proteins present on cellular membranes (4–6). β_2_-glycoprotein I (β_2_ GP1) present on circulating cells such as platelets, monocytes, and endothelial cells is believed to be the primary target. Murine models of APS show that mice treated with anti-β_2_ GP1 antibodies become more susceptible to thrombosis upon vascular injury (7). Multiple broader pathophysiologic mechanisms have been proposed in APS, including activation of platelets, endothelial cells, monocytes and neutrophils, direct activation of coagulation, inhibition of natural anticoagulant systems, and complement activation (4, 5, 8–10). *In vitro* studies have indicated possible activation by aPL of toll-like receptors (TLR2, TLR4) on monocytes, neutrophils, platelets and endothelial cells, leading to thromboinflammation (11–14). However, it remains unclear which cells in the circulation, and what inflammatory pathways, are the primary targets of aPL (4).

Proteome profiling may aid in answering some of these questions and has become a valuable tool in understanding pathophysiology and facilitating biomarker discovery. Proteomics is especially valuable for determining the activity of transcriptomically quiescent cells, such as neutrophils and platelets. Plasma proteomics has been valuable in understanding inflammatory responses to infections such as COVID-19, rheumatologic diseases, diabetes, liver disease and Alzheimer’s dementia (15–18). Seeking to define the inflammatory signature of APS and to enable biomarker discovery, we carried out an unbiased proteomic screen of patients with different APS subtypes, and validated our findings in a second cohort. We found that the mere presence of aPL triggers multiple thromboinflammatory pathways characterized by activation of complement, coagulation, and both innate and adaptive immune systems. Furthermore, the clinical spectrum of APS, from NoAPS to MAPS/CAPS (catastrophic antiphospholipid syndrome), represents a progression of these thromboinflammatory pathways culminating in extracellular matrix (ECM) involvement and tissue inflammation.

## Material and methods

### Samples procurement and study approval

**Figure 1:**
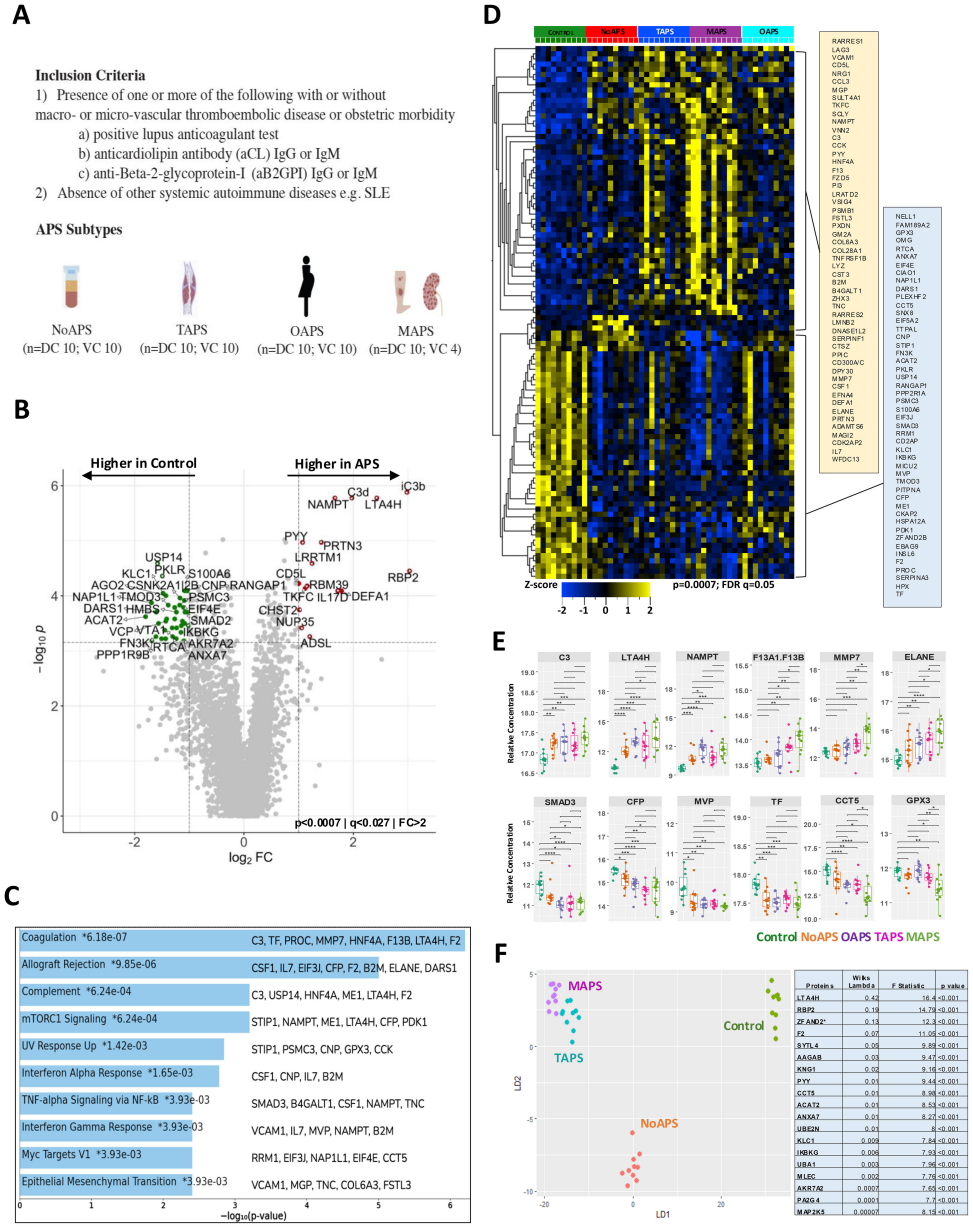
Presence of antiphospholipid antibodies confers a distinct proteomic signature shared by all APS subtypes. **(A)** Study cohorts and inclusion criteria. APS subtypes: NoAPS, aPL positivity without thromboembolism/obstetric morbidity; TAPS, history of thromboembolism, not meeting criteria for microvascular APS; OAPS, history of obstetric morbidity only; MAPS, biopsy-proven microvascular APS or history of definite catastrophic APS (61). n, sample size; DC, discovery cohort; VC, validation cohort. **(B)** Volcano plot of differentially abundant proteins between controls and all APS combined (*p* < 0.0007; *q* < 0.027; fold-change (FC)>2). All proteins are shown. Red dots, proteins higher in APS; green dots, proteins higher in controls. **(C)** Top 10 pathways enriched (KEGG 2021) in APS derived from protein list in *B*, along with the corresponding *p-*values. Asterisk (*) denotes that the term also has a adjusted *p* < 0.005. **(E)** Select differentially abundant proteins (C3, LTA4H, NAMPT, F13, MMP7, ELANE, SMAD3, CFP, MVP, TF, CCT5, GPX3); FDR-corrected t-test on log_2_-normalized data; ****p<0.0001; ***p<0.001; **p<0.01; *p<0.05. **(D)** Z-score heatmap of differentially abundant proteins (rows; table on right) between different APS subtypes and controls (columns); ANOVA, *p ≤* 0.0007, FDR *q ≤* 0.05. **(F)** Unsupervised clustering of non-obstetric APS and controls by linear discriminant analysis (proteins tabulated on right together with Wilks lambda, *F*-statistic and *p*-value).

Plasma samples were obtained from APS ACTION registry, an international and collaborative clinical registry and repository of blood samples. All patients included in this study were persistently aPL-positive (measured on two occasions 12 weeks apart) and without systemic autoimmune rheumatic diseases (SARDs) (19). Samples were not obtained during the time of the event, but at the time of outpatient referral/enrollment. Samples were split into discovery (North American Centers) and validation (European Centers) cohorts (**Figure 1**, **Table 1**).

**Table 1:** Demographic, clinical, and laboratory characteristics of APS patients and controls.

	No APS	OAPS only	TAPS +/- OAPS	MAPS* +/- OAPS/TAPS	Control
Discovery cohort (n: 40)					
Number of Patients	10	10	10	10	10
Demographics					
- Mean Age (registry entry)	46 +	42 + 14	48 + 12	40 + 15	43 + 8
- Female	12	10	5	4	7
- White	9 7	10	10	8	8
Clinical manifestations (ever)					
- Arterial Thrombosis	N/A	N/A	5	3	N/A
- Venous Thrombosis	N/A	N/A	6	7	N/A
- Microvascular Disease**	N/A	N/A	N/A	10	N/A
- Pregnancy Morbidity***	N/A	10	2	0	N/A
- Thrombocytopenia****	0	2	4	7	N/A
- Hemolytic Anemia*****	0	0	1	2	N/A
- Heart Valve Disease	1/8	0/7	0/10	1/8	N/A
aPL characteristics******					
- Triple (LA, aCL, and aβ_2_GPI)	10	6	10	7	N/A N/A
- Double including LA	0	0	0	2	N/A
- Double excluding LA	0	3	0	0	N/A
- Single (LA)	0	1	0	1	N/A
Medications (Baseline)					
- Aspirin	8	6	4	5	N/A
- Warfarin	1	1	8	7	N/A
- Low-molecular-weight-heparin	0	1	0	0	N/A N/A
- Hydroxychloroquine	4	4	4	7	N/A
- Statin	1	1	2	4	N/A
Validation cohort (n: 34)					
Number of Patients	10	10	10	4	
Demographics					
- Mean Age (registry entry)	44 +	38 + 6	40 + 12	60 + 8	
- Female	13	10	5	3	
- White	7 8	10	10	3	
Clinical manifestations					
- Arterial Thrombosis	N/A	N/A	3	4	
- Venous Thrombosis	N/A	N/A	7	0	
- Microvascular Disease*	N/A	N/A	N/A	4	
- Pregnancy Morbidity***	N/A	10	1	1	
- Thrombocytopenia****	4	0	2	0	
- Hemolytic Anemia*****	1	1	0	0	
- Heart Valve Disease	2/8	0/10	1/10	1/4	
aPL characteristics******					
- Triple (LA, aCL, and aβ_2_GPI)	6	3	5	1	
- Double including LA	3	0	3	0	
- Double excluding LA	0	1	0	0	
- Single (LA)	1	4	2	3	
- Single (aCL or aβ_2_GPI)	0	2	0	0	
Medications (Baseline)					
- Aspirin	6	9	4	4	
- Warfarin	0	0	7	2	
- Low-molecular-weight-heparin	0	1	0	0	
- Hydroxychloroquine	6	7	4	0	
- Statin	1	0	3	3	

APS, antiphospholipid syndrome; aPL, antiphospholipid antibodies; aCL, anticardiolipin antibodies; aβ2GPI, anti-β2-glycoprotein-I antibodies; LA, lupus anticoagulant test; NoAPS, aPL only, no clinical APS; OAPS, obstetric APS; TAPS, thrombotic APS; and MAPS, microvascular APS. N/A, Not applicable.*Two patients with catastrophic APS classification in the discovery cohort and none in the validation cohort; **Microvascular disease defined as renal, skin, pulmonary, and/or brain involvement; ***Based on the Revised Sapporo APS classification criteria definitions; ****Thrombocytopenia defined as a platelet count of <100 x 10^9^/L tested twice at least 12 weeks apart; *****Autoimmune hemolytic anemia defined as persistent anemia with hemolysis and a positive direct antiglobulin test; and ******Positive aCL/aβ_2_GPI defined as persistent (at least 12 weeks apart) IgG (+/- IgM) levels >40 Units (enzyme-linked immunosorbent assay) except four patients who had only aCL/aβ_2_GPI IgM levels >40 Units.

Control subjects were healthy volunteers with no history of thromboembolism, SARD, cancer, or anticoagulant/antiplatelet medication use, enrolled from healthcare employees at hematology clinic at Yale New Haven Hospital (**Table 1**).

The protocol was approved by the Institutional Review Boards of all participating institutions. Informed consent was obtained from all participants. Blood samples were collected in EDTA tubes and processed within two hours.

### SomaScan assay

We measured plasma proteins using SomaScan assay, a technology based on highly-specific single-stranded DNA aptamers, which quantifies relative concentrations of plasma proteins (20). The characteristics, sensitivity/specificity and reproducibility of SomaScan to human targets have been previously described (20–23). 6398 unique proteins were measured in the discovery cohort using SomaLogic 7K assay. Subsequently, 1500 proteins were measured in validation cohort using same platform. Median CV between two runs was 4.3% with 90% of analytes having a CV <8.7%.

### Data analysis

All values were technically valid and above the limit-of-detection and there were no missing values (24). First, raw data was log_2_-normalized. To identify differentially abundant proteins between two groups we used t-test; in case of more than two comparators we used ANOVA. In each case, we applied Benjamini-Hochberg correction for multiple comparisons to control the false discovery rate (FDR) at least *q* < 0.1 or *q* < 0.5, thereby minimizing type I errors inherent in simultaneous testing of thousands of proteins. Protein abundance was considered different when the test of statistical significance was met at the specified *p*- and *q*-values. We used hierarchical clustering and z-score heat maps (normalized to a mean of 0, variance of 1) to visualize differential proteins, which were considered upregulated or downregulated if the difference was at least 1.5-fold. These analyses were carried out in Qlucore Omics Explorer (Lund, Sweden). Box, volcano, and radar plots were generated with ggplot2, EnhancedVolcano, and ggradar packages, respectively, in R/RStudio (R Core Team, Vienna, Austria/RStudio Team, Boston, MA).

For linear discriminant analysis (LDA), we implemented a two-stage feature selection approach to address the high-dimensional nature of the proteomics data (p >> n). First, we generated candidate differential proteins using the Wilcoxon rank-sum test with FDR adjustment at q<0.2, chosen as a dimension reduction strategy to retain potentially informative features while mitigating multicollinearity. Second, we applied stepwise forward variable selection using greedy Wilk’s Lambda criterion to select proteins based on their discriminatory capacity. The LDA procedure projects high-dimensional protein data onto 2–3 linear discriminants (depending on group number), with each subject’s position determined by linear discriminant scores to enable visualization and classification. This analysis was performed using MASS and klaR packages in R.

### Functional enrichment analysis

We utilized gene annotation and analysis resources for functional enrichment analysis has been previously done (15, 16, 18). We specifically used Enrichr and Metascape suites for this analysis (25, 26).

## Results

### aPL confer a distinct plasma proteomic signature shared by all APS subtypes

We performed an unbiased plasma proteomic screen in 40 persistently aPL-positive patients: 10 each with NoAPS, TAPS, MAPS, or OAPS (**Figure 1A**). The median age was 48 years, with 70% females. Approximately 70% were ‘triple-positive’ (i.e., positive for lupus anticoagulant, anticardiolipin antibodies IgG/M ‗ 40U, and anti-β_2_ GP1 IgG/M ‗ 40U), none with a concomitant diagnosis of SARDs. The clinical characteristics of the cohort are described in **Table 1**. Ten additional healthy adults, without any history of thrombosis, SARD or cancer, were enrolled as controls.

We measured 6398 unique plasma proteins in all 50 samples by SomaLogic 7K assay (20). First, to characterize broadly plasma proteomic abnormalities in APS, we compared all APS subtypes combined versus controls. The volcano plot in **Figure 1B** highlights the differentially abundant proteins between the two groups. A comprehensive list of all measured plasma proteins, difference between control and APS, and relevant statistics are provided in **Supplementary Table 1**. The most significantly differential proteins have been discussed in greater detail below including in subsequent analyses.

Next, we carried out functional enrichment analysis from the list of differentially abundant proteins obtained above, to identify the implicated biological pathways (**Figure 1C**, **Supplementary Table 2**). The most significant pathways were Coagulation (C3, tissue factor, protein C, MMP7, HNF4A, LTAH4, and prothrombin), Allograft Rejection (CSF1, IL7, EIF3J, CFP, prothrombin, B2M, ELANE, DARS1) and Complement (C3, USP14, HNF4A, ME1, LTA4H, prothrombin). Additionally, alterations in Interferon, IL-6/JAK/STAT3, PI3K/AKT/mTOR, and ILT-STAT4 signaling pathways highlight the thromboinflammatory milieu that defines APS, including NoAPS.

Following this, we compared each of the four APS subtypes with healthy controls using ANOVA. This analysis revealed a proteomic signature shared by all APS subtypes, including NoAPS group, as compared with controls (**Figure 1D**). There was heterogeneity among APS patients, even within the same categories, but despite this, different APS subtypes were hierarchically clustered. The differentially abundant proteins are listed next to the heat map. Generally, the z-scores for the differentially abundant proteins altered in APS were more significant in TAPS, but particularly in MAPS subtype, suggesting that plasma thromboinflammatory abnormalities in APS reflect APS severity. OAPS appeared distinct, which was somewhat unexpected (27, 28). For subsequent analyses, OAPS was explored separately.

To study proteomic effects, if any, of treatments, we carried out analysis stratified on vitamin K antagonists (15 out of 20 on warfarin), hydroxychloroquine (9 out of 20 on hydroxychloroquine), statins (8 out of 20 on statins) and aspirin (9 out of 20 on aspirin) in TAPS and MAPS combined (**Supplementary Figure S2**). As expected, Vitamin K-dependent proteins were found to be lower in individuals on VKA (**Supplementary Figures S2A, B****),** but proteomic abnormalities in hydroxychloroquine group could only be detected at very high false discovery rates (**Supplementary Figures S2C, D**). Individuals on statin had proteomic abnormalities that included higher insulin-like growth factor binding protein 1 (IGFBP1) and two members of sialic-acid binding immunoglobulin-like lectins (SIGLECs) (**Supplementary Figures S2E, F**). There were no significant abnormalities noted in aspirin group (**Supplementary Figure S2G**). None of the treatments affected proteomic abnormalities previously found to be associated with APS.

Several proteins displayed a positive trend across the phenotypes from NoAPS to MAPS (**Figure 1E**, top panel), while others showed a negative trend (**Figure 1E**, lower panel), suggesting an association with clinical severity of APS. Notably, tissue factor (TF) and complement factor P (CFP), a positive regulator of the alternative complement pathway, were lower in APS, particularly in MAPS. Patients with a ‘triple-positive’ aPL profile are known to be at risk for subsequent events. Our data suggests that NoAPS can be associated with a protein profile linked to thromboinflammation, which is shared across APS subtypes (29).

Next, we attempted unsupervised clustering using linear discriminant analysis to identify proteins that would classify individual samples to their phenotypes (NoAPS, TAPS, MAPS) and controls. Indeed, LDA distinctly separated NoAPS, TAPS and MAPS from controls (**Figure 1F**). The set of discriminant proteins with corresponding discriminatory metric (Wilk’s lambda) is shown in the table and discussed in a greater detail below; proteins with lower Wilk’s lambda are more discriminatory.

### Proteomic abnormalities associated with aPL

**Figure 2:**
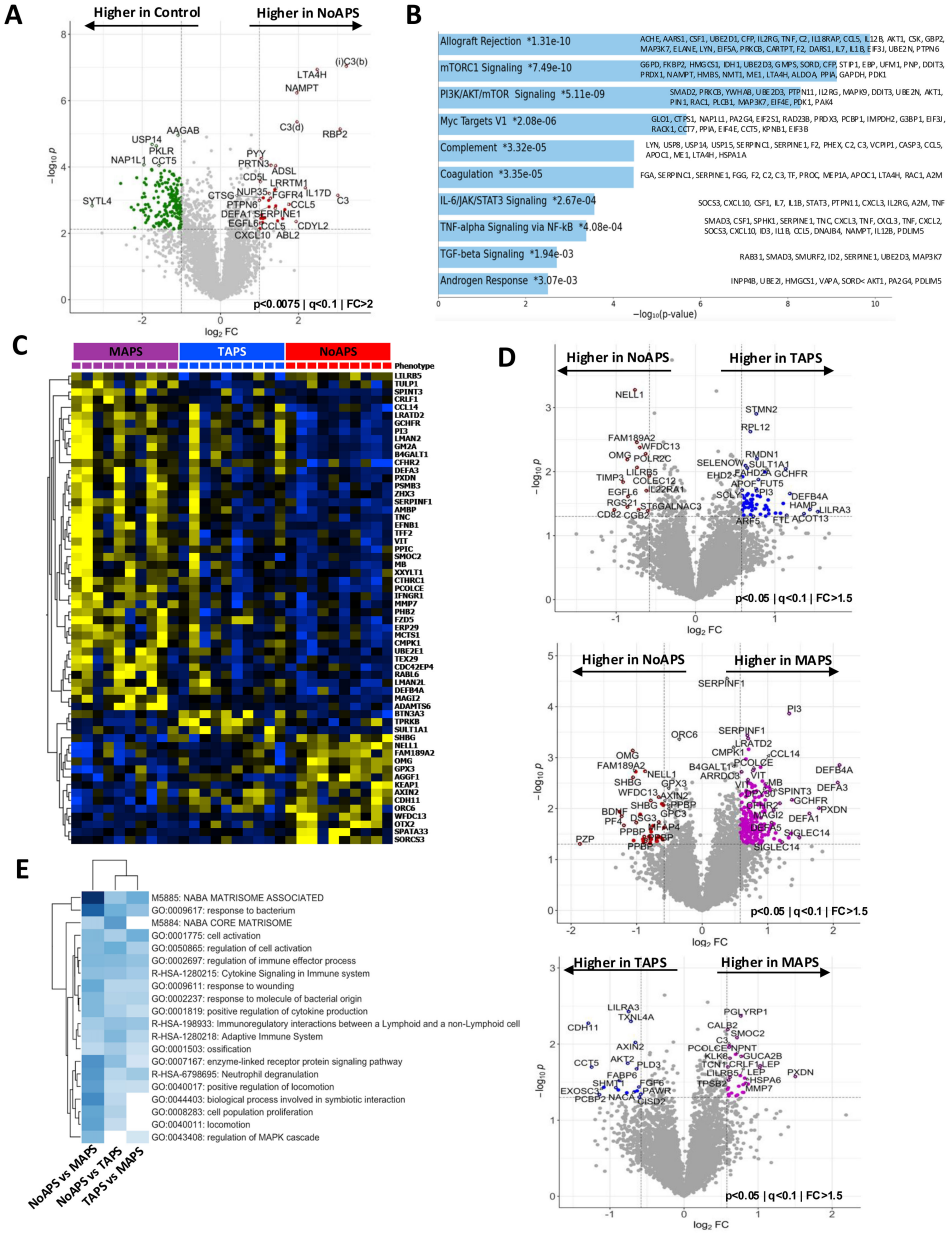
Plasma proteomic abnormalities associated with the ‘first-hit’; increasing thromboinflammation in TAPS and MAPS. **(A)** Volcano plot of differentially abundant proteins between NoAPS and controls (*p* < 0.0075; *q* < 0.1; fold-change (FC)>2). All proteins are shown. Red dots, proteins significantly higher in aPL; green dots, proteins significantly higher in controls. **(B)** Bar chart showing top 10 enriched terms (KEGG 2021) in the protein list generated by the pairwise analysis of NoAPS vs control in *A*, along with the corresponding *p-*values. Asterisk (*) denotes that the term also has a *p* < 0.005). **(C)** Z-score heatmap of differentially abundant proteins (rows; tabulated on right) between NoAPS, TAPS and MAPS (columns); ANOVA, *p ≤* 0.0007, FDR *q ≤* 0.1. **(D)** Volcano plots of differentially abundant proteins derived from three individual pairwise analyses between NoAPS and TAPS (top), NoAPS and MAPS (middle), and TAPS and MAPS (bottom) (*p* < 0.05; i<0.1; fold-change (FC)>1.5). All proteins are shown. Colored dots depicting differentially abundant proteins, directionality shown on the plots. **(E)** Heatmap showing the top 20 enriched terms derived from the protein sets (*p* < 0.05) obtained from the pairwise analyses between NoAPS and TAPS, NoAPS and MAPS and TAPS and MAPS in D.

The ‘two-hit’ model of APS posits that vascular injury or inflammation (“second hit”) triggers thromboembolism in a circulation previously primed by aPL (“first hit”) (5). Given the significant plasma proteomic changes in NoAPS cohort seen above (**Figures 1B, C**), we wanted to further characterize the thromboinflammatory milieu associated with this “first hit”. Therefore, we performed pairwise analysis between NoAPS and controls. The most significant differences were in C3, RBP2, LTA4H, NAMPT, IL17D, CCL5, and CXCL2 (high in NoAPS), as well as SYTL4, ARHGAP45, INPP5B and PRKCB (low in NoAPS) (**Figure 2A**). Hierarchical clustering of differentially abundant proteins obtained in this analysis also showed a clear distinction between NoAPS and controls (**Supplementary Figure S1**). Functional enrichment analysis again highlighted alterations in complement, coagulation, innate immunity, IL6 as well as TNF-alpha signaling pathways, among others (**Figure 2B**). This, we propose, is the thromboinflammatory signature of the “first hit” of APS. Interestingly, Human Phenotype Ontology identified multiple thromboembolism phenotypes using the set of proteins differentially abundant between NoAPS and controls (**Supplementary Figure S1B**). Pairwise comparisons of controls with TAPS or MAPS also identified similar protein sets, particularly C3, LTA4H, NAMPT, PRTN3, and RBP2, (**Supplementary Figure S1C**).

C3 and other complement abnormalities are well established in APS and have also been targeted therapeutically (4). Leukotriene A4H (LTA4H), which was also one of the top hits in LDA (**Figure 1E**), although primarily synthesized by liver, is released by many circulating immune cells, including neutrophils, and there is evolving literature on the role of LTA4H in inflammatory diseases and preclinical data on therapeutic utility of LTA4H inhibitors (30, 31). Circulating NAMPT, acting as a damage associated membrane protein (DAMP), is a well-known regulator of inflammation specifically through activation of pattern recognition receptors toll-like receptors (TLRs) such as TLR4 (32, 33). Isoforms of RBP have also been identified as proinflammatory mediators in conditions such as type 2 diabetes mellitus (32, 33).

Our data clearly show that many of the inflammatory pathways recognized in the context of severe clinical APS subtypes, are already active in asymptomatic individuals with circulating aPL.

### From laboratory APS to thromboembolic disease: proteomic abnormalities in thrombotic APS

Identification of pathophysiologic abnormalities associated with development of clinical APS from NoAPS would allow for risk stratification of APS but also potentially novel therapeutic biomarkers. We hypothesized that this would manifest through differences in proteomic profiles of varying APS subtypes and performed pairwise analyses between NoAPS and TAPS, NoAPS and MAPS, and TAPS and MAPS cohorts.

First, hierarchical clustering of differentially abundant proteins obtained from multigroup ANOVA showed a clear distinction across NoAPS, TAPS, and MAPS (**Figure 2C**). A trend between APS severity and plasma proteomic abnormality, in both the number of differentially abundant proteins and z scores, was again noted, with MAPS having the greatest number of alterations, while TAPS appearing intermediate. The volcano plots highlight the most significant differentially abundant proteins between NoAPS and TAPS, NoAPS and MAPS, and TAPS and MAPS (**Figure 2D**). Again, the number of differentially abundant proteins (defined by adjusted *p*-value <0.05) was highest in the NoAPS vs. MAPS (440), as compared with NoAPS vs. TAPS (225), and TAPS vs. MAPS (190), with many proteins being common (116 proteins) (**Supplementary Table 3**). This suggests that plasma proteomic alterations increase with APS severity.

Lastly, we performed functional enrichment analyses using Metascape suite, which allowed us to compare the three proteins sets obtained from the pairwise comparisons above (NoAPS vs MAPS, NoAPS vs TAPS and TAPS vs MAPS) (**Figure 2E**). Among the top 20 functional pathways identified and shared between the three groups, the most significant differences were identified in NoAPS vs. MAPS, based on the z scores. This analysis provides insights into the inflammatory responses in APS beyond that of laboratory APS. Although neutrophil degranulation, cell signaling and proliferation, cytokine signaling, and pathways related to innate and adaptive immune systems were activated, the most significant changes were seen in pathways connected to ECM organization. Increasing strength of alterations in coagulation pathway was also notable. In summary, our data reveals a pattern of progressive thromboinflammation with increasing clinical APS severity.

### Microvascular antiphospholipid syndrome is characterized by increasing thromboinflammation and extracellular matrix reorganization

Organ or life-threatening multisystemic thromboembolic disease is a hallmark of MAPS/CAPS. Infections as well as individual level risk factors such as inherited complement factor abnormalities have been identified as provoking factors in some individuals, but what triggers these severe clinical manifestations of APS remains largely undefined (4, 34, 35). Therefore, we attempted to identify biologic pathways that distinguish MAPS from TAPS.

**Figure 3:**
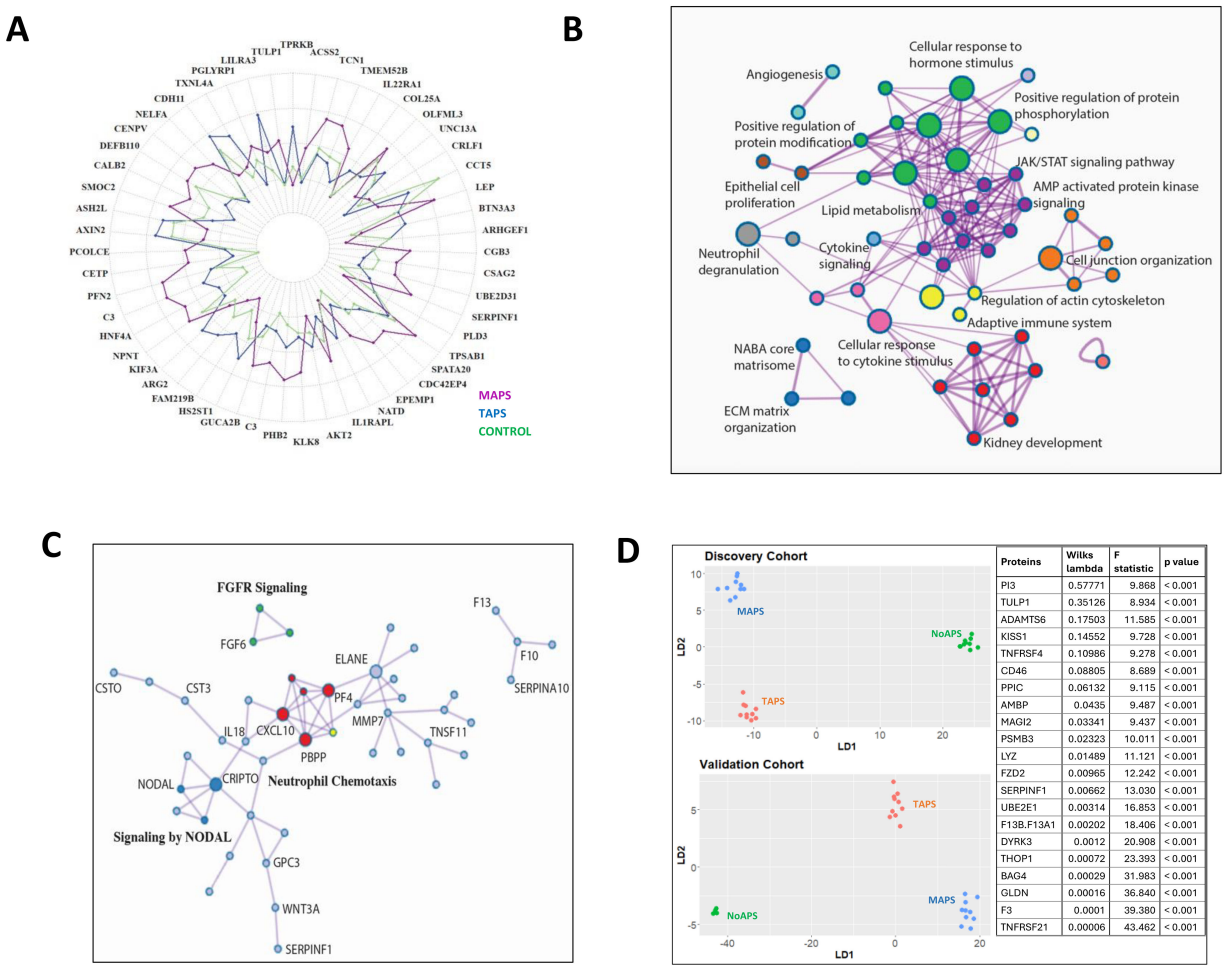
Thromboinflammation resulting in extracellular matrix reorganization in MAPS **(A)** Radial plot of the top 50 differentially abundant proteins comparing MAPS (purple) with TAPS (blue); relative protein abundance in controls (green) shown as baseline. **(B)** Network plot of enriched terms obtained from differential protein list from TAPS vs. MAPS comparison (**Figure 2D**). Terms with a similarity of >0.3 are connected by edges. Each node represents an enriched term and is colored by its cluster ID. The most significant of the enriched terms are labeled in the network plot. **(C)** Protein-protein interaction enrichment analysis of 85 proteins belonging to ECM-associated pathways complied from the pairwise analyses between aPL or TAPS and MAPS. Network contains proteins that form physical interactions with at least one other member in the list. Pathway enrichment analysis applied to the most densely connected network components. Most significant terms include neutrophil chemotaxis (red), FGFR signaling (green), and NODAL pathways (dark blue). **(D)** Linear discriminant analysis/unsupervised clustering of NoAPS, TAPS and MAPS in validation cohort (bottom plot) using protein set generated by linear discriminant analysis in discovery cohort (top plot) (outlined in the panel on right together with Wilks lambda, F-statistic and p-value).

The pairwise comparison between TAPS and MAPS identified differentially abundant proteins that could distinctly cluster the two cohorts (**Figure 2D**, **Supplementary Figure S1D**). In **Figure 3A**, the relative mean protein levels of 50 top hits are shown as a radar plot; 34 proteins were higher in MAPS than TAPS. To further understand the functional nature of these proteomic changes, we created a network plot of biologic pathways identified from this protein list (adjusted *p*-value < 0.05) (**Figure 3B**). Many of the pathways identified, specifically intracellular signaling pathways, vesicular trafficking, protein phosphorylation, angiogenesis, and metabolism, extend beyond the abnormalities already noted in NoAPS and TAPS.

Notably, ECM abnormalities and many kidney-associated pathways suggest increasing tissue inflammation in MAPS. The role of ECM in inflammation and thrombosis, is well established (36, 37). The proteins belonging to the ECM-related pathways, compiled from both TAPS vs. MAPS and NoAPS vs. MAPS analyses, included proteases (neutrophil elastase (ELANE), cathepsins G (CTSG), O (CTSO), and V (CTSV)), protease inhibitors (cystatins B (CYTB) and C (CTS3), serine proteinase inhibitors (SERPIN) B8, B9, A7, A10 and F1; peptidase inhibitor 3 (PI3) and PZP alpha-2-macroglobulin like (PZP)); metalloproteinases (matrix metallopeptidase 7 (MMP7), pappalysin 1 (PAPPA), ADAMTS4, 6 and 13), metalloproteinase inhibitor TIMP3, growth factors (fibroblast growth factors (FGF) 6, 9, and 20; platelet derived growth factor (PDGF); growth differentiation factors (GDF) 2 and 15; and angiopoietin 2 (ANGPT2)), glycans and glycan-binding receptors (glypicans (GPC) 1 and 5; C-type lectin receptors (CLEC) 3B, 4G and 9A; collectin subfamily member 12 (COLEC12)), and cytokines CXCL10, CCL16, interleukin 7 (IL7), IL17, IL19; and TNF superfamily members (TNFSF) 11 and 12. Protein-protein interaction enrichment analysis of these 85 ECM-associated proteins was performed; the accompanying plot highlights the most densely connected network components underscoring the role of ECM proteins in neutrophil chemotaxis. chemokine-mediated signaling, FGF activation and NODAL pathways in MAPS (**Figure 3C**). Overall, the involvement of ECM, metabolism, intracellular signaling and trafficking pathways, signify the depth of MAPS-associated inflammation.

### Validation

We sought to validate these findings in an additional cohort comprising of 34 individuals (10 NoAPS, 10 TAPS, 4 MAPS, and 10 OAPS; **Table 1**). Other than the geographic location, there were no differences in the clinical characteristics between the two cohorts. For validation, we estimated the 1500 most differentially abundant proteins identified in the original analysis using the same assay (**Supplementary Table 4**). We were able to obtain hierarchical clustering of different APS subtypes in the validation cohort (**Supplementary Figure S3**).

Next, we wanted to see if a single set of discriminant proteins could cluster NoAPS, TAPS and MAPS in both the cohorts. For this, we first repeated linear discriminant analysis of the discovery cohort with the 1500 proteins measured in the validation cohort (**Figure 3D** top). Subsequently, we attempted clustering of the validation cohort by this protein set. Notably, the same set of discriminant proteins identified from the discovery cohort, clustered NoAPS, TAPS and MAPS in validation cohort (**Figure 3D** bottom). The proteins featured in this analysis (table on the right) included cytokines (TNFRSF21 and TNFRSF4), TNF receptor signal transducers (BAG4, DYRK3) coagulation factors (tissue factor, factor 13), proteases (lysozyme), protease inhibitors (PI3), and ECM regulators (ADAMTS6), that play a role in innate and adaptive immunity, thrombosis, ECM organization, and regulation of tissue inflammation.

### Obstetric antiphospholipid syndrome

**Figure 4:**
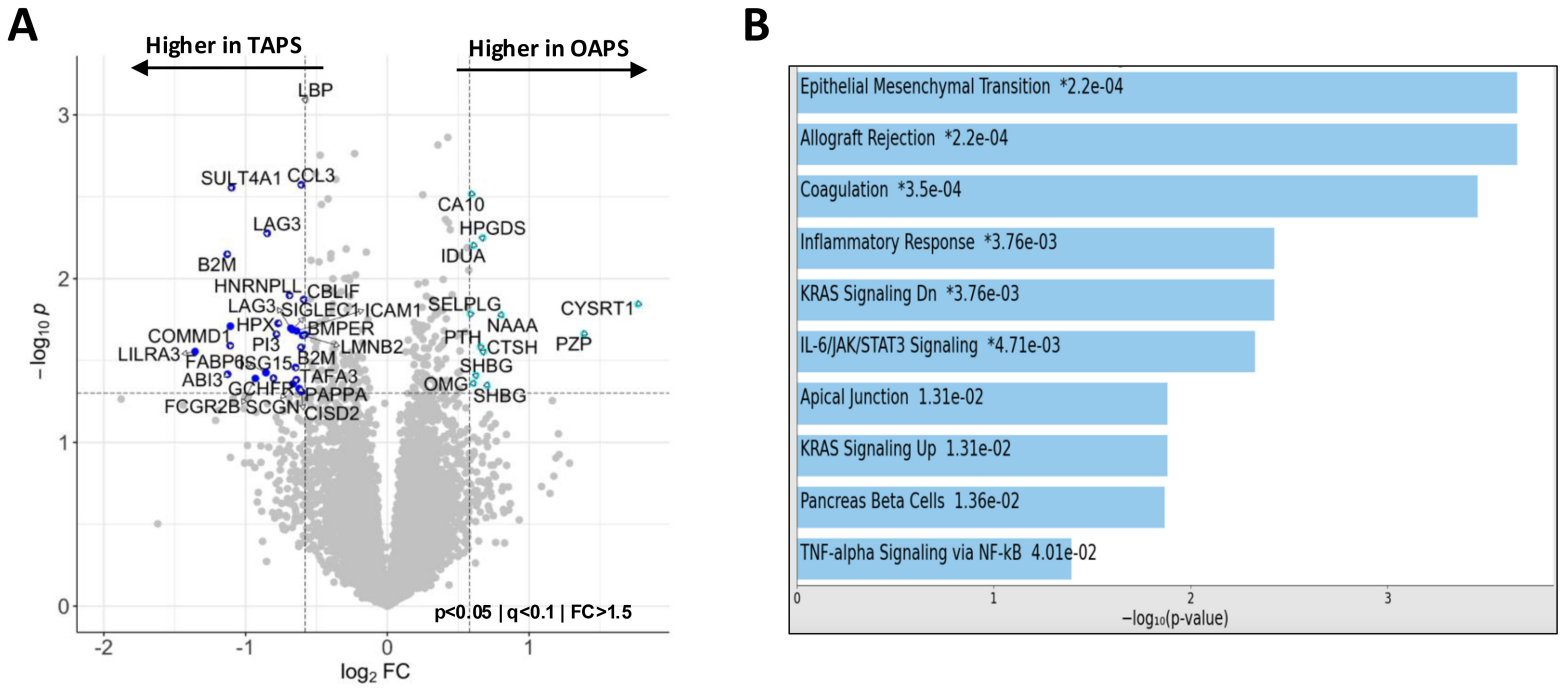
Plasma proteomics abnormalities in OAPS. **(A)** Volcano plot of differentially abundant proteins between TAPS and OAPS (p<0.05; q<0.1; fold change (FC)>1.5). **(B)** Bar chart showing the top 10 enriched terms (KEGG 2021 Human Library) in the protein list generated by the pairwise analysis of TAPS vs OAPS in *A*, along with the corresponding *P* values. Asterisk (*) denotes that the term also has a significant adjusted *P* value (<0.005).

Although OAPS and NoAPS shared many of the proteomic alterations, individuals with a history of OAPS appeared distinct from TAPS and MAPS (**Figure 1B**). To evaluate plasma proteomic changes that characterize OAPS, first, we compared OAPS with controls. This analysis highlighted previously noted abnormalities shared by all APS subtypes, including C3, LTA4H, RBP2 and NAMPT (**Supplementary Figure S4A**). Next, we compared OAPS and TAPS. The volcano plot in **Figure 4A** shows the differentially abundant proteins between OAPS and TAPS, while **Figure 4B** highlights the functional enrichment analysis of these differential proteins. Restricting this analysis only to females with TAPS (n=7) did not alter the findings (**Supplementary Figure S4B**). Epithelial Mesenchymal Transition (SFRP4, FOXC2, LAMA2, TNC, VIM and FBLN5) was the most significantly altered term including proteins primarily higher in OAPS. Additionally, KRAS Signaling (EGF, GP1BA, PNMT, ITIH3, GDNF) was noted to be distinctive from previous analyses of TAPS and MAPS, also driven by abnormalities in OAPS.

## Discussion

**Figure 5:**
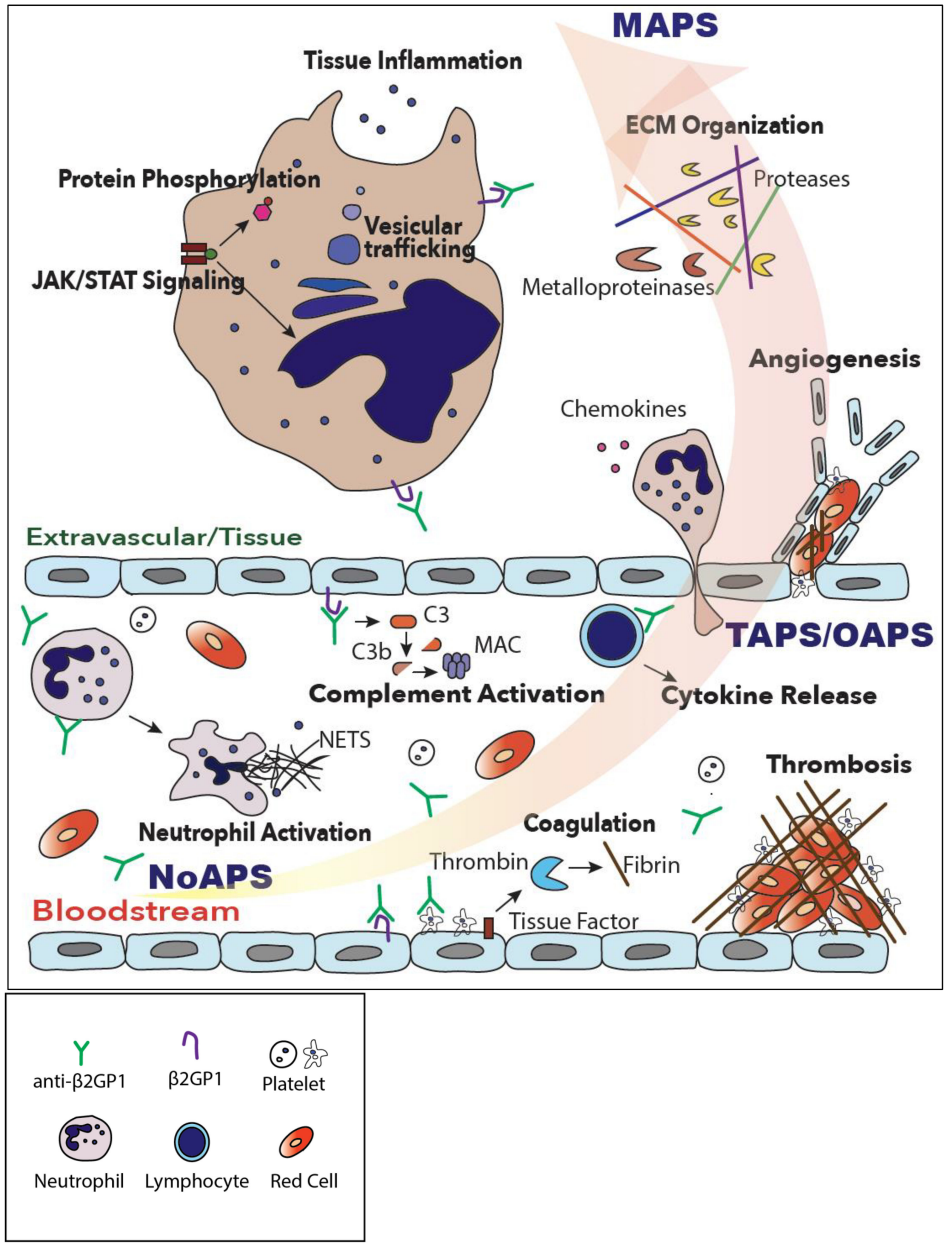
A model of progressive thromboinflammation in primary APS. While complement, coagulation and neutrophil activation are already evident in high-risk, ‘triple-positive’, laboratory APS (NoAPS), clinical subtypes of APS, OAPS, TAPS but particularly microvascular/catastrophic APS (MAPS), are characterized by severe thromboinflammation involving activation of intracellular signaling pathways, vesicular trafficking, protein phosphorylation, tissue inflammation and extracellular matrix organization.

Understanding molecular mechanisms underlying APS could facilitate biomarker discovery for both diagnostic as well as therapeutic purposes. With this goal, we carried out comprehensive plasma proteomic profiling of patients with four different subtypes of primary APS (laboratory, thrombotic, obstetric, and microvascular/catastrophic) using a highly sensitive and specific single stranded-DNA aptamer-based assay (20, 21). Despite heterogeneity among APS patients, our data show that mere presence of circulating aPL induces multiple inflammatory pathways, particularly in individuals with ‘high-risk’ phenotype, a signature shared by all APS subtypes and consistent with the “first hit” of APS. Subsequently, the evolution of APS from laboratory to thrombotic and especially microvascular/catastrophic disease is characterized by increasing activation of coagulation and complement systems, native and adaptive immunity, culminating in ECM organization and tissue inflammation (**Figure 5**).

Coagulation functional pathway abnormalities were identified as one of the principal proteomic abnormalities in APS. These proteins included procoagulants such as tissue factor, fibrinogen, factor XIII, as well as natural anticoagulants such as antithrombin, but also proteins related to pathobiology of coagulation such as complement proteins (C3), metalloproteinases (MMP7) and other serine protease inhibitors (SERPINE1 or PAI-1). Notably, we found reduce levels of plasma TF in APS, which could either reflect consumption and clearance of circulating soluble TF or elevated membrane binding. Similarly, CFP was lower in APS, which could reflect consumption or compensatory regulation of this positive regulator of complement pathway. We also identified abnormalities in factor II (prothrombin), and protein C, but these vitamin K-dependent proteins could have been affected by warfarin that some subjects were taking for anticoagulation. Additionally, complement proteins including C3 and complement regulators such as CFP, CD55, CD46, HNF4A, were differentially abundant in APS. Complement abnormalities have been well known in APS and have also been exploited therapeutically in treatment of MAPS (34). More importantly, our data provide evidence of progressive and overlapping activation of coagulation and complement pathways in development of thrombotic and microvascular forms of APS.

Some of the proteins that were highly significant and discriminatory in severe types of APS included LTA4H, NAMPT, TNFRSF21, TF, SULT4A1, which can potentially be used as biomarkers, both diagnostic, for risk stratification of APS, as well as therapeutic targets. In particular, LTA4H and NAMPT, which are known to play a role in pathophysiology of many inflammatory disorders, including SLE, and LTA4H has also been investigated as a potential therapeutic target (30–33). NAMPT, a DAMP, activates TLRs, which are known to play a critical role in pathophysiology of APS (4). TNFRSF21 (also known as death receptor 6 (DR6), a member of TNF receptor superfamily, is involved in immune regulation through NF-kB and has also been shown to be important in pathophysiology of many inflammatory disorders, including SLE (38).

Our findings also support a central role of immune activation in APS. Several immunologically relevant pathways were found to be activated encompassing both innate and adaptive immune systems. The most significant of these involve neutrophils (neutrophil activation, degranulation, and extracellular trap formation), phagocytosis activation, natural killer cell activation, platelet activation, and related pathways such as cytokine and interleukin signaling, JAK/STAT activation, and tyrosine kinase receptor signaling. Neutrophils have previously been reported to play an important role in pathophysiology of APS (4, 5, 10, 39). It remains unclear, though, whether neutrophils are the primary target of aPL or if neutrophil activation occurs as a secondary event. Anti-β2GP1, the pathologic antibodies implicated in APS, have been reported to activate multiple cellular components in the circulation, including platelets, endothelium, and neutrophils (4, 5, 11, 12, 40). Specifically, antibody-β2GP1 complex has been shown to interact and activate surface receptors such as glycoprotein 1bα, LRP8, TLRs. Our proteomics data also implicate activation of TLR, with evidence of activation of both TLR4-MyD88 and TLR4-TRIF pathways, as evidenced by the differentially abundant MAPK2K3, MAP3K7, BTK, IKBKG, PTPN11, BIRC2, SKP1, and UBE2 isoforms, particularly in MAPS (41). Our data also aligns with recent transcriptomics studies in APS, which includes bulk RNA sequencing or microarray analysis of whole blood, neutrophils, aortic valve tissue, and primary human endothelial cells treated with IgG fraction from APS patients (42–47). These studies identified and highlighted a role for interferon regulated genes and signaling (IFITs, IFNL3 etc.), cytokines (IL2, IL2R, IL6, IL15 etc.), chemokines (CCL13, CXCL10 etc.), other genes mediating both innate and adaptive immune responses (CYP26B1, LIFR, NLRPs, TRAF3, LILRA and B etc.), as well as ECM proteins (MMPs, SERPINs etc.), found to be significant in our proteomics analysis.

Proteomic abnormalities in TAPS and particularly MAPS provide evidence of both increasing intensity of thromboinflammation and cellular and tissue inflammation. This is evidenced by proteins belonging to the ECM organization, as well as intracellular vesicular, lysosome, and membrane trafficking, receptor mediated endocytosis, Rab GTPase activation, and various intracellular signaling pathways and metabolism. Notably, a growing body of literature supports a critical role played by ECM proteins in pathogenesis of systemic inflammatory diseases and chronic diseases that are known to occur through vascular and immune pathways (36, 37, 48, 49). We also found many plasma proteins in MAPS belonging to kidney-associated pathways, of particular interest in light of the kidney as a primary disease site in MAPS (3).

The proteomic alterations in OAPS appeared distinct, particularly when compared to TAPS and MAPS. This may reflect the modulation of immunoinflammatory milieu known to be associated with pregnancy (27, 28). Unlike other tissues, β2GP1 is known to have a high constitutive expression in extra-villous trophoblastic and syncytio-trophoblastic cells of the placenta, and may be essential for normal pregnancy (50–52). Could this differentially regulate the effect of anti-2GP1 antibodies and account for OAPS to have a distinct proteomic signature we noted? Moreover, regulated complement activation may be required for normal placentation, although dysregulated complement activation is a hallmark of APS as well as that of other pregnancy complications, such as pre-eclampsia (53). Given the role of β2GP1 as a complement regulator, it’s interaction with complement pathway may affect APS in pregnancy distinctively (54). These will require evaluation of larger cohorts of patients with OAPS, as well as further mechanistic studies.

Our study has some limitations. First, our sample size was relatively modest, but the consistency of our findings in the two cohorts provides meaningful insights that warrant further validation in larger cohorts of APS. Moreover, controls were not matched for age and sex, although the reproducibility of the observed patterns across the two demographically different cohorts supports the robustness of the most significant proteomics abnormalities in APS. While our data provide a comprehensive analysis of plasma proteome of APS and its subtypes, the exact mechanisms by which the identified inflammatory pathways evolve from aPL to either TAPS or MAPS remain unknown. Moreover, the most significant APS-associated proteins identified here need to be validated by specific immunoassays, which we did not perform. Notably, previously a high correlation between SomaScan and immunoassays has been reported (55, 56). Second, SomaScan 7K assay is known to contain an over-representation of secreted proteins. Mass spectrometry (MS)-based proteomics would be an ideal method for unbiased screening but carries low sensitivity. In fact, MS-based proteomics has previously been attempted on plasma, urine and extracellular vesicles in APS and a systematic review of 11 studies yielded only a combined 82 dysregulated proteins, belonging to cellular activation/degranulation and thrombosis pathways (57–60). Next, our samples were not drawn at the time of thromboembolic event. Although, the proteomic abnormalities we report may reflect the ‘baseline inflammation” associated with different APS subtypes, inducible proteins expected to be expressed only at the time of clinical events and relevant to thrombosis may be missed. Moreover, the trend in identified proteomic abnormalities is not known and it is not possible to exclude reverse causation, that is, some of the proteins may be a consequence of disease manifestation. The possibility of false positives also exists with simultaneous testing of thousands of proteins, but we used standard statistical approaches to control the false discovery rate, thereby minimizing type 1 errors. Lastly, our studies do not provide any evidence of the implicated upstream pathways or inflammatory cells directly involved in APS initiation. Despite these limitations, we provide the most comprehensive and up to date proteomic analysis of APS and its subtypes, and our findings provide important insights into the two-hit model of APS and identify potential new targets for risk stratification and therapeutic intervention in APS across the entire spectrum of clinical disease.

## Data Availability

The raw data supporting the conclusions of this article will be made available by the authors, without undue reservation.
