# Correction: A proteomic map of thromboinflammatory signatures in antiphospholipid syndrome: results from antiphospholipid syndrome alliance for clinical trials and international networking (APS ACTION) registry

**DOI:** 10.3389/fimmu.2026.1807468

**Published:** 2026-04-10

**Authors:** Alexander Pine, Ayesha Butt, Laura Andreoli, Jason S. Knight, Maria Gerosa, Irene Cecchi, D. Ware Branch, Rosario Lopez-Pedrera, H. Michael Belmont, Nina Kello, Michelle Petri, Ricard Cervera, Vittorio Pengo, Pier Luigi Meroni, Hannah Cohen, Rohan Willis, Maria Laura Bertolaccini, George Goshua, Sean Gu, John Hwa, Alfred I. Lee, Doruk Erkan, Anish V. Sharda

**Affiliations:** 1 Section of Hematology, Yale University School of Medicine, New Haven, CT, United States; 2 University of Brescia, Brescia, Italy; 3 University of Michigan, Ann Arbor, MI, United States; 4 University of Milan, Milan, Italy; 5 University of Turin, Turin, Italy; 6 University of Utah and Intermountain Healthcare, Salt Lake, UT, United States; 7 Maimonides Institute of Biomedical Research of Cordoba, Córdoba, Spain; 8 New York University Langone Medical Center, New York, NY, United States; 9 Northwell Health, New Hyde Park, NY, United States; 10 Johns Hopkins University School of Medicine, Baltimore, MD, United States; 11 Department of Autoimmune Diseases, Hospital Clínic, Institut d’Investigacions Biomèdiques August Pi i Sunyer (IDIBAPS), University of Barcelona, Barcelona, Catalonia, Spain; 12 University of Padova, Padova, Italy; 13 IRCCS Istituto Auxologico Italiano, Milan, Italy; 14 University College London, London, United Kingdom; 15 University of Texas Medical, Galveston, TX, United States; 16 St Thomas’ Hospital, London, United Kingdom; 17 Yale Cardiovascular Research Center, Yale University School of Medicine, New Haven, CT, United States; 18 Hospital for Special Surgery and Weill Cornell Medicine, New York, NY, United States

**Keywords:** antiphospholid syndrome, plasma proteomics, thromboinflammation, immune activation, coagulation, complement

Author Pier Luigi Meroni was erroneously assigned to affiliation “University of Milan, Milan, Italy”. This affiliation has now been updated to “IRCCS Istituto Auxologico Italiano Milan, Italy.”

The original version of this article has now been updated.

