# Supplementary material for: A proteomic map of thromboinflammatory signatures in antiphospholipid syndrome: results from antiphospholipid syndrome alliance for clinical trials and international networking (APS ACTION) registry
Source: Front Immunol. 2025 Oct 16;16:1676578. doi: 10.3389/fimmu.2025.1676578 (PMC12571811; doi:10.3389/fimmu.2025.1676578)
Supplement: Supplementary file 1 [file DataSheet1.pdf]

Figure S1

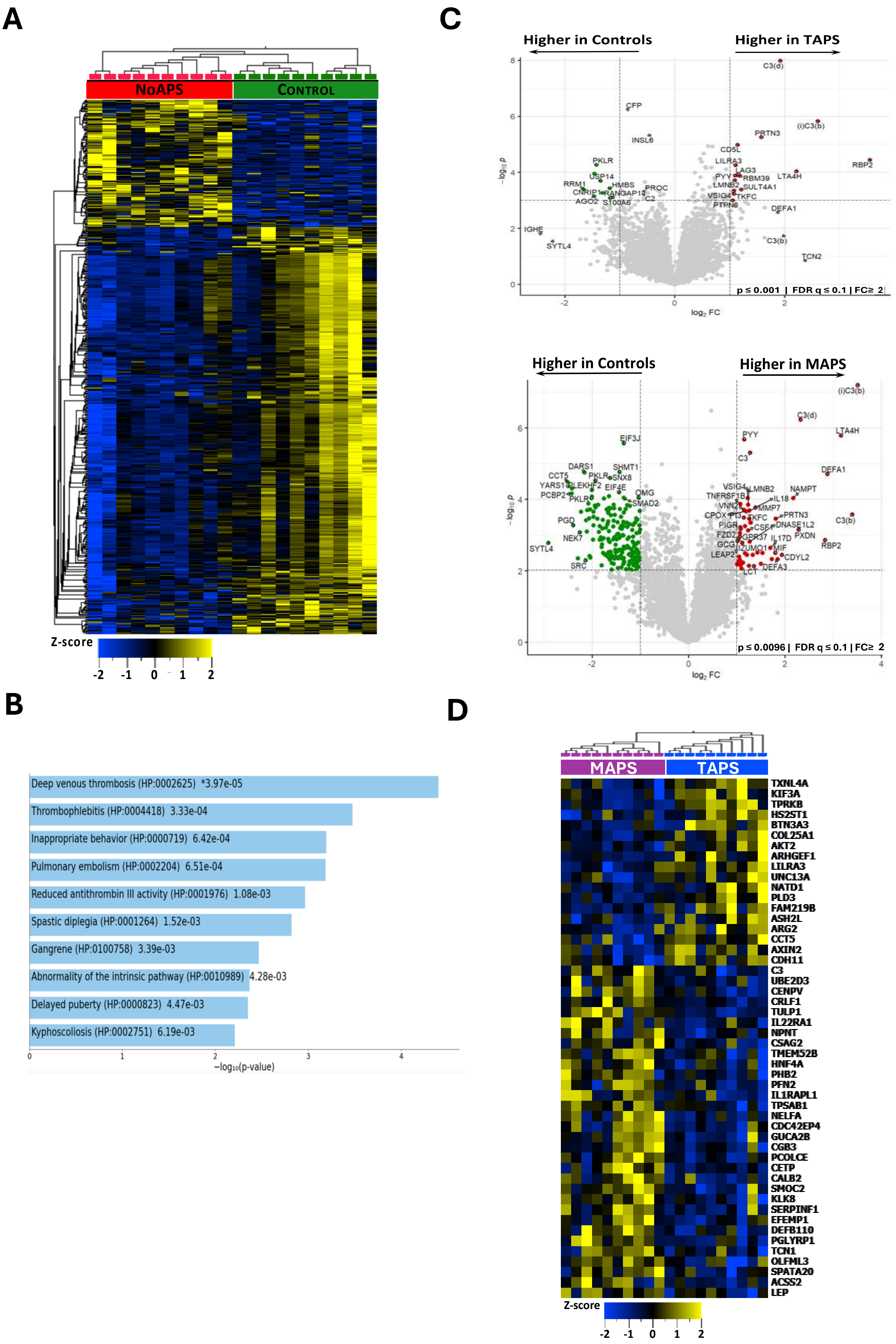
A
NoAPS
Control
Z-score
-2 -1 0 1 2
B
Deep venous thrombosis (HP:0002625) *3.97e-05
Thrombophlebitis (HP:0004418) 3.33e-04
Inappropriate behavior (HP:0000719) 6.42e-04
Pulmonary embolism (HP:0002204) 6.51e-04
Reduced antithrombin III activity (HP:0001976) 1.08e-03
Spastic diplegia (HP:0001264) 1.52e-03
Gangrene (HP:0100758) 3.39e-03
Abnormality of the intrinsic pathway (HP:0010989) 4.28e-03
Delayed puberty (HP:0000823) 4.47e-03
Kyphoscoliosis (HP:0002751) 6.19e-03
0 1 2 3 4
−log₁₀(p-value)
C
Higher in Controls
Higher in TAPS
p ≤ 0.001 | FDR q ≤ 0.1 | FC≥ 2
log₂ FC
−log₁₀ p
Higher in Controls
Higher in MAPS
p ≤ 0.0096 | FDR q ≤ 0.1 | FC≥ 2
log₂ FC
−log₁₀ p
D
MAPS
TAPS
TXNL4A
KIF3A
TPRKB
HS2ST1
BTN3A3
COL25A1
AKT2
ARHGEF1
LILRA3
UNC13A
NATD1
PLD3
FAM219B
ASH2L
ARG2
CCT5
AXIN2
CDH11
C3
UBE2D3
CENPV
CRLF1
TULP1
IL22RA1
NPNT
CSAG2
TMEM52B
HNF4A
PHB2
PFN2
IL1RAPL1
TPSAB1
NELFA
CDC42EP4
GUCA2B
CGB3
PCOLCE
CETP
CALB2
SMOC2
KLK8
SERPINF1
EFEMP1
DEFB110
PGLYRP1
TCN1
OLFML3
SPATA20
ACSS2
LEP
Z-score
-2 -1 0 1 2

Figure S2

A

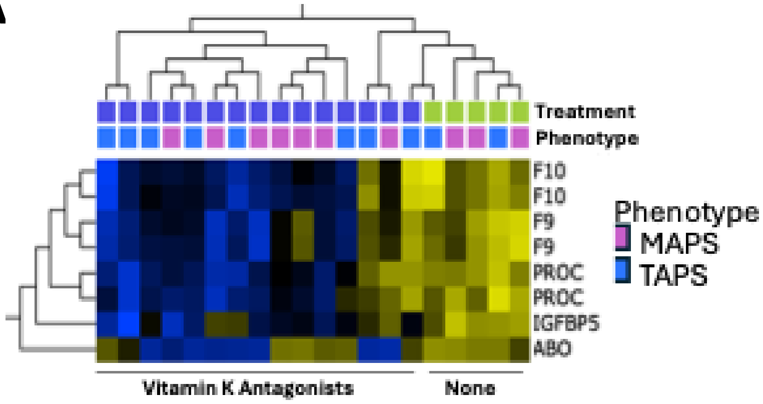

p≤0.0005; q≤0.38

Gene	Target	q-value
PROC	Activated Protein C	0.06112
IGFBP5	IGFBP-5	0.0875
F10	Coagulation Factor Xa	0.1654
PROC	Protein C	0.1654
F10	Coagulation Factor X	0.3180
ABO	BGAT	0.3180
F9	Coagulation Factor IXab	0.3518
F9	Coagulation Factor IX	0.3850

B

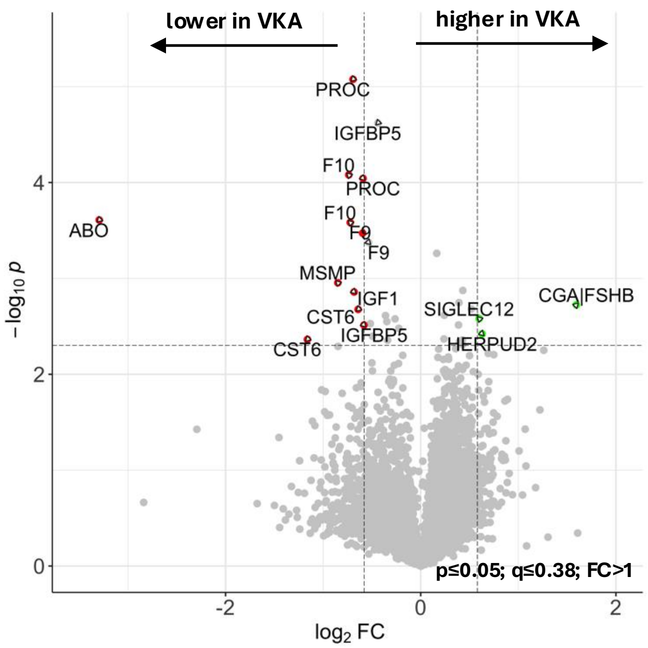

C

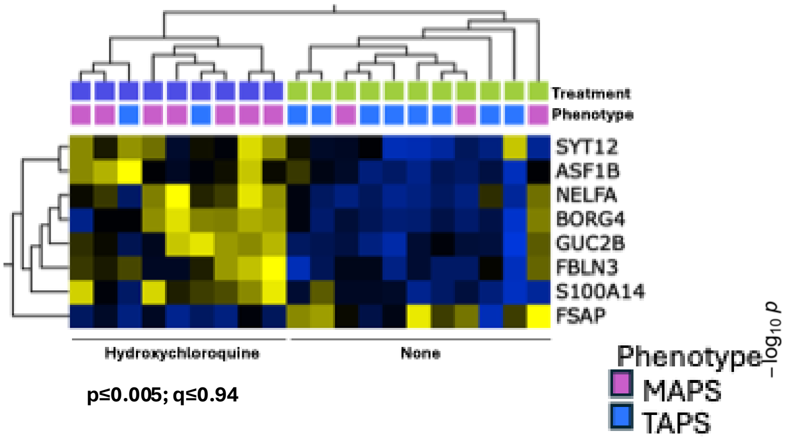

p≤0.005; q≤0.94

Gene	q-value
SYT12	0.9419
ASF1B	0.9419
NELFA	0.9419
CDC42EP4	0.9419
GUCA2B	0.9419
EFEMP1	0.9419
S100A14	0.9419
HABP2	0.9419

D

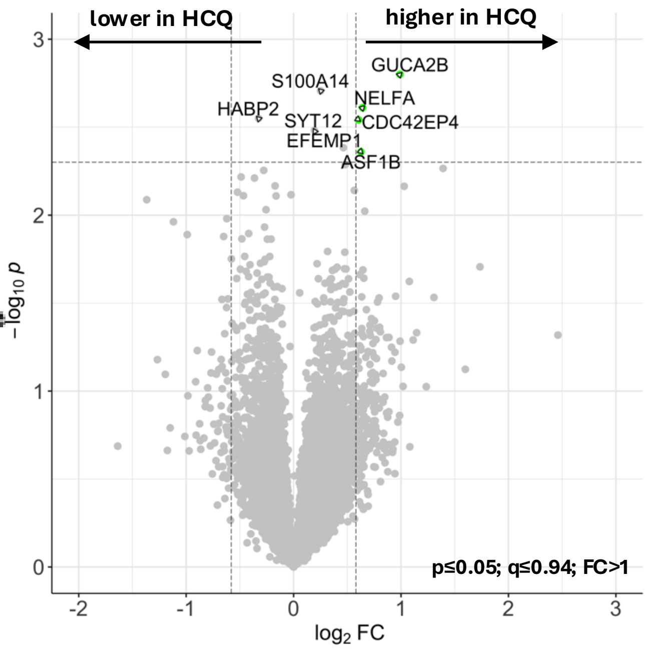

Figure S2

E

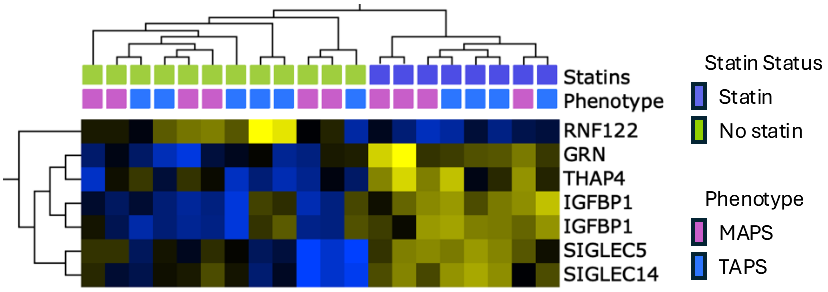

p≤0.005; q≤0.47

Gene	p-value	q-value	log_2FC
IGFBP1	2.23E-05	0.1628	2.24
IGFBP1	7.19E-05	0.2267	2.09
GRN	9.32E-05	0.2267	0.48
THAP4	0.0003	0.4406	1.12
SIGLEC5	0.0004	0.4406	0.69
RNF122	0.0004	0.4406	-0.34
SIGLEC14	0.0005	0.4587	0.73

F

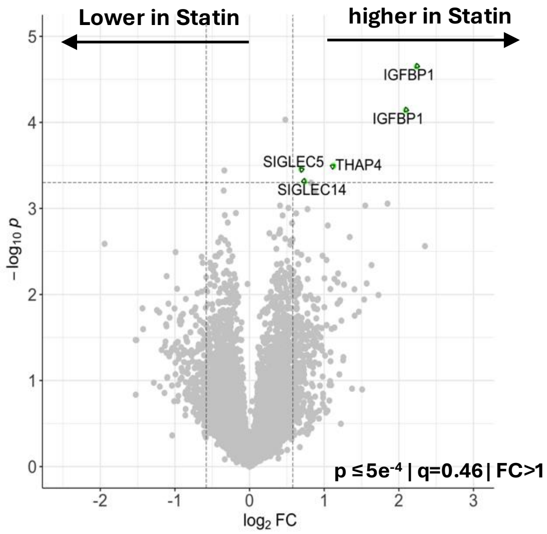

G

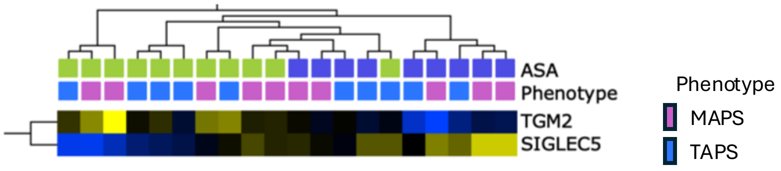

p≤0.005; q≤0.95

Figure S3

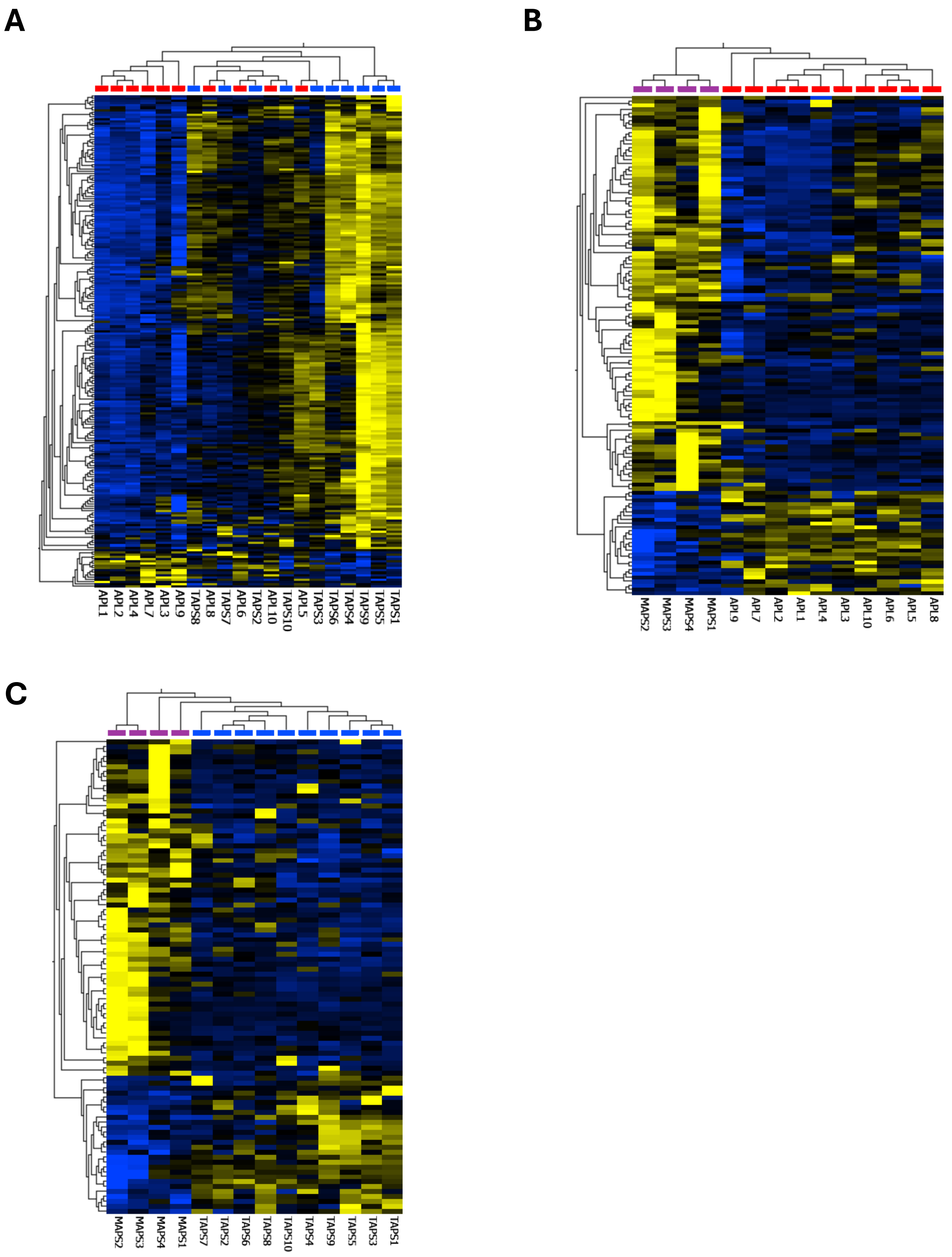
A
APL1
APL2
APL4
APL7
APL3
APL9
TAPS8
APL8
TAPS7
APL6
TAPS2
APL10
TAPS10
APL5
TAPS3
TAPS6
TAPS4
TAPS9
TAPS5
TAPS1
B
MAPS2
MAPS3
MAPS4
MAPS1
APL9
APL7
APL2
APL1
APL4
APL3
APL10
APL6
APL5
APL8
C
MAPS2
MAPS3
MAPS4
MAPS1
TAPS7
TAPS2
TAPS6
TAPS8
TAPS10
TAPS4
TAPS9
TAPS5
TAPS3
TAPS1

Figure S4

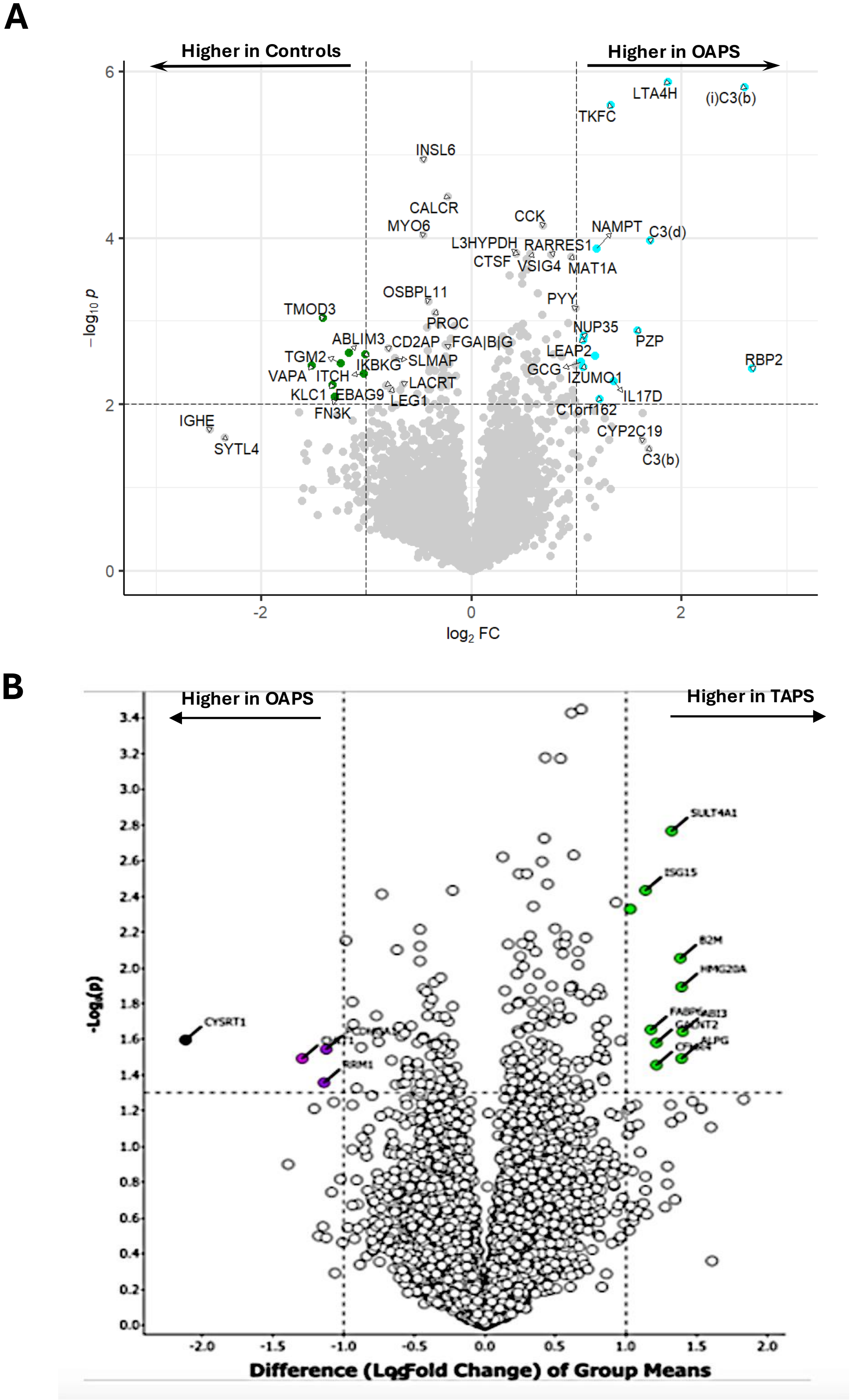
A
Higher in Controls
Higher in OAPS
LTA4H
(i)C3(b)
TKFC
INSL6
CALCR
MYO6
CCK
NAMPT
C3(d)
L3HYPDH
RARRES1
CTSF
VSIG4
MAT1A
OSBPL11
PYY
TMOD3
PROC
NUP35
PZP
ABLIM3
CD2AP
FGA|B|G
LEAP2
RBP2
TGM2
IKBKG
SLMAP
GCG
IZUMO1
VAPA
ITCH
LACRT
KLC1
EBAG9
LEG1
IL17D
FN3K
C1orf162
IGHE
CYP2C19
SYTL4
C3(b)
−log10 p
log2 FC
B
Higher in OAPS
Higher in TAPS
SULT4A1
ISG15
B2M
HMG20A
ALPG
CYSRT1
RRM1
-Log10(p)
Difference (Log2 Fold Change) of Group Means
