# Supplementary material for: A proteomic map of thromboinflammatory signatures in antiphospholipid syndrome: results from antiphospholipid syndrome alliance for clinical trials and international networking (APS ACTION) registry
Source: Front Immunol. 2025 Oct 16;16:1676578. doi: 10.3389/fimmu.2025.1676578 (PMC12571811; doi:10.3389/fimmu.2025.1676578)
Supplement: Supplementary file 2 [file DataSheet2.docx]

**Supplementary Figure Legends**

**Figure S1.** (A) Z-score heatmap of differentially abundant proteins (rows) between individuals with NoAPS compared with controls (columns); Pairwise analysis with *p*≤0.0075, FDR *q*≤0.1. (B) Bar chart showing the top 10 enriched terms (Human Phenotype Ontology) in the protein list generated by the pairwise analysis between NoAPS and control. Asterisk (*) denotes that the term also has a significant adjusted *p*<0.005. (C) Volcano plot of differentially abundant proteins between TAPS and controls (top) and MAPS and controls (bottom) (*p*<0.001; *q*<0.1; fold-change (FC)>2). All proteins are shown, with red dots depicting differentially abundant protein significantly higher in TAPS or MAPS and green dots significantly higher in controls. (D) Z-score heatmap of differentially abundant proteins (rows; tabulated on right) between individuals with TAPS and MAPS (columns); Pairwise analysis with *p*≤0.02 estimated to account for the false discovery rate with *q*≤0.1.

**Figure S2. Stratification by treatment.** (A) Z-score heatmap of differentially abundant proteins (rows) in individuals with TAPS and MAPS stratified on vitamin K antagonists (VKA) treatment (columns); Pairwise analysis with *p*≤0.0005, FDR *q*≤0.38. Table (below) lists differential proteins with corresponding *q* statistic (B) Volcano plot for the analysis in A. (C) Z-score heatmap of differentially abundant proteins (rows) in individuals with TAPS and MAPS stratified on hydroxychloroquine treatment (columns); Pairwise analysis with *p*≤0.005, FDR *q*≤0.94. Table (below) lists differential proteins with corresponding *q* statistic (D) Volcano plot corresponding to C. (E) Z-score heatmap of differentially abundant proteins (rows) in individuals with TAPS and MAPS stratified on statin treatment (columns); Pairwise analysis with *p*≤0.005, FDR *q*≤0.46. Table (below) lists differential proteins with corresponding *p ,q* and fold-change statistics (F) Volcano plot for analysis in E. (G) Z-score heatmap of differentially abundant proteins (rows) in individuals with TAPS and MAPS stratified on aspirin treatment (columns); Pairwise analysis with *p*≤0.005, FDR *q*≤0.95.

**Figure S3. Pairwise analyses in validation cohort**. (A-C) Heatmap showing differentially expressed proteins comparing NoAPS vs TAPS (A), NoAPS vs MAPS (B) and TAPS vs MAPS (C). Linear models were fit at each protein level with the phenotype being tested as the main effect (*p*,0.05;FDR<0.1). Each row represents expression of a single protein, while each cell represents the Z score of a protein expressed in a study subject.

**Figure S4. Pairwise comparison between (A) OAPS and controls and (B) OAPS and TAPS (females only).** Volcano plot showing differentially expressed proteins between OAPS and controls (A) and OAPS and TAPS (females only) (B). Linear models were fit at each protein level with OAPS as the main effect (*p*<0.01;FDR<0.1).

**Supplementary Table Legends**

**Supplementary Table 1.** Table showing all measured plasma proteins comparing controls vs All APS combined.

**Supplementary Table 2.** Table showing all Hallmark 2020 pathways based on differentially abundant proteins identified in ANOVA between control and different APS subtypes.

**Supplementary Table 3.** Table showing differentially abundant proteins between three pairwise analyses in figure 3. The proteins in red font are present in at least 2 pairwise analyses.

**Supplementary Table 4.** List of proteins measured in the validation cohort.
